# Proceedings of the second international meeting on endemic mycoses of the Americas (IMEMA) and first international symposium on implantation mycoses (ISIM)

**DOI:** 10.1093/mmy/myae054

**Published:** 2024-05-14

**Authors:** Norma B Fernandez, Diego H Cáceres, Julian A Serrano, Alexandro Bonifaz, Cristina E Canteros, Roberto Suarez-Alvarez, Rosely Maria Zancope Oliveira, Regielly C R Cognialli, Priscila Marques de Macedo, Beatriz L Gomez, Angela M Tobon, Carlos Taborda, Tom Chiller, Jose Guillermo Pereira Brunelli, Dallas J Smith, Marcus de Melo Teixeira, Flavio Queiroz-Telles, Guillermo Garcia-Effron, Karina Ardizzoli, Ricardo Negroni, Gustavo Giusiano

**Affiliations:** Sección Micologia, División de Infectología, Hospital de Clínicas José de San Martin, Buenos Aires, Argentina; Micología Clínica. Facultad de Farmacia y Bioquímica. Universidad de Buenos Aires, Ciudad Autónoma de Buenos Aires, Argentina; Studies in Translational Microbiology and Emerging Diseases (MICROS) Research Group, School of Medicine and Health Sciences, Universidad del Rosario, Bogota, Colombia; Center of Expertise in Mycology Radboudumc/Canisius Wilhelmina Hospital, SZ Nijmegen, The Netherlands; IMMY, Norman, Oklahoma, USA; Sección Micología, Laboratorio Central, Hospital Independencia, Santiago del Estero, Argentina; Dermatology Service, Hospital General de México “Dr. Eduardo Liceaga”, Ciudad de Mexico, México; Departamento Micología, Laboratorio Nacional de Referencia en Micología Clínica, INEI-ANLIS “Dr. Carlos G. Malbrán”, Ciudad Autónoma de Buenos Aires, Argentina; Laboratorio de Colecciones de Cultivos Microbianos del INEI-ANLIS “Dr. Carlos G. Malbrán”, Ciudad Autónoma de Buenos Aires, Argentina; Laboratório de Micologia, Instituto Nacional de Infectologia Evandro Chagas, Fundação Oswaldo Cruz, Rio de Janeiro, Brazil; Mycology Unit, Hospital de Clinicas, Federal University of Paraná, Curitiba, Brazil; Laboratory of Clinical Research on Infectious Dermatology, Instituto Nacional de Infectologia Evandro Chagas, Fundação Oswaldo Cruz., Rio de Janeiro, Brazil; Studies in Translational Microbiology and Emerging Diseases (MICROS) Research Group, School of Medicine and Health Sciences, Universidad del Rosario, Bogota, Colombia; Instituto Colombiano de Medicina Tropical, Universidad CES, Medellín, Colombia; Department of Microbiology, Biomedical Sciences Institute University of São Paulo, São Paulo, Brazil; Laboratory of Medical Mycology, Institute of Tropical Medicine of São Paulo LIM53/Medical School, University of São Paulo, São Paulo, Brazil; Mycotic Diseases Branch, Centers for Disease Control and Prevention, Atlanta, United States of America; Centro de Especialidades Dermatologicas. Ministerio de Salud Pública y Bienestar Social de Paraguay, San Lorenzo, Paraguay; Mycotic Diseases Branch, Centers for Disease Control and Prevention, Atlanta, United States of America; Faculty of Medicine, University of Brasília, Brasília, Brazil; Department of Public Health, Hospital de Clinicas, Federal University of Parana, Curitiba, Brazil; Laboratorio de Micología y Diagnóstico Molecular - Cátedra de Parasitología y Micología - Facultad de Bioquímica y Ciencias Biológicas - Universidad Nacional del Litoral, Santa Fe, Argentina; Consejo Nacional de Investigaciones Científicas y Técnicas (CONICET), Ciudad Autónoma de Buenos Aires, Argentina; Sector Micología, Servicio de Laboratorio, Hospital Interzonal de Agudos, Dr. R. Rossi, La Plata, Buenos Aires, Argentina; Micología Clínica, Facultad de Ciencias Exactas, Universidad Nacional de La Plata, La Plata, Buenos Aires, Argentina; Adviser of Mycology Unit, Francisco J. Muñiz Hospital, Ciudad Autónoma de Buenos Aires, Argentina; Instituto de Medicina Regional, Universidad Nacional del Nordeste, Consejo Nacional de Investigaciones Científicas y Técnicas de Argentina (CONICET), Resistencia, Argentina

**Keywords:** Blastomycosis, coccidioidomycosis, histoplasmosis, paracoccidioidomycosis, sporotrichosis, chromoblastomycosis, mycetoma

## Abstract

The second international meeting on endemic mycoses of the Americas (IMEMA) and the first international symposium on implantation mycoses (ISIM) took place in Santiago del Estero, Argentina, on September 25–27, 2023. The conference provided a platform for researchers, clinicians, and experts to discuss the latest developments in the field of endemic and implantation mycoses. Topics included epidemiology, diagnostic advances, treatment strategies, and the impact of environmental factors on the spread of these fungal diseases. IMEMA and ISIM contributed to the regional discourse on the mycoses, emphasizing the importance of international cooperation in addressing these public health challenges.

## Second IMEMA and first ISIM

The second international meeting on endemic mycoses of the Americas (IMEMA) and the first international symposium on implantation mycoses (ISIM) convened in Santiago del Estero, Argentina, during September 25–27, 2023, with more than 100 attendees. The meeting was a pivotal gathering for the exchange of knowledge and advancements in the field of endemic and implantation mycoses. With a One Health focus, the conference brought together experts, researchers, and healthcare professionals from across the Americas to discuss critical aspects of these mycoses. The meeting facilitated a comprehensive discussion on various aspects of the following fungal infections: blastomycosis, coccidioidomycosis, histoplasmosis, paracoccidioidomycosis (PCM), sporotrichosis, chromoblastomycosis, and mycetoma. In addition, the meeting covered relevant aspects related to taxonomical updates on etiological agents of systemic and implantation mycoses and included discussion of atypical clinical presentations and differential diagnoses.

Most of these mycoses are considered sapronotic, i.e., the etiological agent is acquired from the environment where the fungus fulfills part of its life cycle. The infection occurs by inhalation of contaminated aerosolized soils, most frequently in blastomycosis, coccidioidomycosis, histoplasmosis, and PCM, or by traumatic implantation through the skin, mostly in sporotrichosis, chromoblastomycosis, and mycetoma.^1,2^ Sporotrichosis has also emerged as an epizootic disease since 1998, that is, it may be sapro-zoonoses.^3–5^ These mycoses have caused outbreaks, linked directly or indirectly to ecological variations as consequences of certain human activities including construction, migration, climatic events, tourism, and wars; outbreaks have also been associated with global climate anomalies and natural disasters such as dust storms, hurricanes, floods, and landslides.^6,7^

Blastomycosis is caused by fungi of the genus *Blastomyces*. In the Americas, cases have been mostly reported in North America, in geographic areas surrounding the Ohio and Mississippi River valleys, and in the Great Lakes region. In general, blastomycosis has a low incidence with less than two cases per 100 000 in the population, but in hyper-endemic areas, such as Wisconsin in the United States (US), where incidence can be as high as 10–40 cases per 100 000.^8,9^

Coccidioidomycosis, caused by *Coccidioides immitis* and *Coccidioides posadasii*, is endemic in the regions of the southwestern US, in the states of Washington and North Mexico, in some regions in Guatemala, Honduras, Venezuela, northeast Brazil, west Paraguay and south Bolivia, and in the arid pre-Andes region of Argentina. In 2019, the US Centers for Disease Control and Prevention (CDC) reported 20 003 cases; most were reported in the states of Arizona and California. The burden of coccidioidomycosis is unknown in most regions where the disease is endemic, due to the lack of surveillance and reportable status.^10,11^

In recent years, the concept of histoplasmosis as a disease limited to certain geographic regions in the Americas has changed, and it is now believed to have a global distribution.^12^ In the Americas, histoplasmosis is widely distributed across North, Central, and South America and the Caribbean, except for Chile, where most cases reported have been identified as imported cases.^1,2^ Due to the high prevalence of advanced HIV disease (AHD) in Latin America, histoplasmosis is a highly prevalent opportunistic infection in people living with HIV (PLHIV).^13^ Nonspecific signs and symptoms and a similar presentation as tuberculosis (TB) cause delayed diagnoses. Other immunocompromised patients, such as transplant recipients and people receiving biological and immunosuppressive therapy, are often diagnosed with this disease.^13^

PCM, caused by species belonging to the genus *Paracoccidioides*, is an important systemic mycosis endemic in Latin America. Recently, reclassifications of the genus *Paracoccidioides* and *Lacazia* have been proposed. For the genus *Paracoccidioides*, the proposal included the division of the *P. brasiliensis* species into four species: *P. brasiliensis, P. americana, P. restrepiensis*, and *P. venezuelensis. Paracoccidioides lutzii* did not change. *Lacazia loboi* has been proposed as *Paracoccidioides lobogeorgii* nom. nov. and *Paracoccidioides ceti*.^14^ Both species were considered causative agents of lobomycosis in humans and dolphins; however, molecular taxonomy studies suggested that the former species is the causative agent of the disease in humans and the latter in dolphins. This change involves the classification of the human disease lobomycosis as Jorge Lobo’s disease.^14^ PCM is a disease reported from Mexico to north Argentina, with the highest endemic regions reported in Brazil, Venezuela, Colombia, and Argentina. PCM usually affects individuals who have occupational exposure to soil, such as farmers and agricultural workers, and is most often reported in adult males. In recent decades, an epidemiological shift has been reported in terms of the geographic distribution of PCM in endemic areas of Brazil and Argentina. Some old endemic areas are being replaced by the emergence of new ones due to several factors, including agriculture mechanization, the massive use of azole fungicides for plantations, and natural and anthropic environmental changes, among others.^15–17^ Since PCM is not a notifiable disease in most countries where it is endemic, the burden is unknown, as are most of the mycoses included in IMEMA-ISIM.

Sporotrichosis is an implantation mycosis caused by species of the genus *Sporothrix*, mainly *S. schenckii, S. brasiliensis*, and *S. globosa*. This fungal infection has worldwide distribution, and its estimative annual burden is 40 000 cases.^18^ Historically, the disease was considered acquired through traumatic skin inoculation of the fungus in its mycelial environmental phase.^2–4^ Frequent risk factors include activities that involve handling contaminated soil, plants, or organic matter. Outbreaks have been linked to specific environmental sources, natural disasters, occupations, and recreational activities.^3–5^*Sporothrix brasiliensis* has emerged in the last two decades in Brazil and neighboring countries as a fungal pathogen causing an emerging zoonotic disease transmitted by infected cats.^3–5^

Chromoblastomycosis and mycetoma are chronic deep cutaneous infections caused by the implantation of fungi or bacteria into the skin. It mainly affects the skin and the subcutaneous tissues. These diseases are reported in tropical and subtropical regions around the world. Chromoblastomycosis is commonly caused by several species of melanized (dematiaceous) fungi, including those from the genus *Fonsecaea, Cladophialophora*, and *Phialophora*. Mycetoma is caused by fungi (eumycetoma) and bacteria (actinomycetoma). Eumycetoma is mainly caused by species of the genus *Madurella, Scedosporium, Tremastosphaeria, Fusarium*, and *Falciformispora*. Early recognition of chromoblastomycosis and mycetoma, accurate diagnosis, and proper treatment are crucial for preventing chronic disease, disability, and stigma.^2^ Both implantation mycoses were included as neglected tropical diseases by the World Health Organization (WHO).

## Meeting key findings

Experts shared comprehensive insights on the epidemiology of these diseases, emphasizing country’s expanding endemicity, emerging trends, and factors that influence the spread of these fungal pathogens. This information is crucial for public health planning and intervention strategies. During the meeting, representatives from the Argentinean Ministry of Health shared that all endemic and implantation mycoses are reportable nationally.

Representatives summarized the status of the diagnosis capacity and access for blastomycosis, coccidioidomycosis, histoplasmosis, PCM, sporotrichosis, chromoblastomycosis, and mycetoma (Table 1). In summary, conventional diagnostics based on microscopy and culture are the main methods used to diagnose these diseases. Microscopy and histopathology are rapid and can confirm the diagnosis in most of these mycoses, but have low sensitivity and can vary depending on the experience of the laboratory professionals. Cultures also have low sensitivity, long turn-around times for results, and biohazard risks of handling isolates. In general, antibody tests are well developed, particularly for the diagnosis of systemic endemic mycoses such as coccidioidomycosis, histoplasmosis, and PCM. For implantation mycoses, antibody testing only exists for sporotrichosis and is a useful tool in extra-cutaneous forms of the disease. Currently, antigen testing is only widely available for the diagnosis of histoplasmosis. In theory, detection of 1-3 β-d-glucan (a component of fungal cell wall) could be a useful biomarker, but this type of testing is available in few facilities and not in all countries in the Americas.^19^ Pan-fungal polymerase chain reaction (PCR) testing can detect all these fungal infections, but these methods are only available in specialized laboratories or referral centers.^20^ The shortage of commercial PCR kits based on molecular methods is a critical limitation for the implementation of molecular methodologies outside reference laboratories. The lack of standardization of PCR protocols, and the limited evaluation of the tests on different biological specimens, are also major limitations (Table 1).

**Table 1.** Status of laboratory abilities for the diagnosis of endemic and implantation mycoses.

Mycosis	Microscopy	Culture	Ab^±^	Ag^±^	Molecular	Key notes
**Blastomycosis:** endemic in US and Canada						
In house testing	Yes	Yes*	Yes	No	Yes	Low analytical performance of Ab detection assays.
Commercial testing	No	Yes*	Yes	Yes	No	Ag test is only available for send-out testing in the US.
**Coccidioidomycosis:** endemic in US, Mexico, Guatemala, Honduras, Venezuela, Brazil, Paraguay, Bolivia, and Argentina						
In house	Yes	Yes*	Yes	No	Yes	Most diagnoses are done based on microscopy and Ab detection.
Commercial	No	Yes*	Yes	No	No	Commercial kits are available for Ab detection based on CF, ID, EIA, and LFA.
**Histoplasmosis:** endemic in most the Americas and the Caribbean countries						
In house	Yes	Yes*	Yes	No	Yes	Microscopy is the most useful tool in the diagnosis of patients with cutaneous manifestations.
Commercial	No	Yes	Yes	Yes	Culture identification only	Availability of Ag testing is becoming widely available.EIA and LFA for Ag detection are commercially available, with limited commercialization.Real time PCR and nested PCR assays are available in reference centers.
**Paracoccidioidomycosis:** endemic from South Mexico to North Argentina						
In house	Yes	Yes*	Yes	No	Yes	Microscopy and Ab detection continue to be diagnosed throughout Latin America.Performance of Ab testing varies based on species *P*a*racoccidioides* and autochthonous isolates used for in-house Ag production. No standardized Ag for ID is available.
Commercial	No	Yes	Yes	No	No	Limited availability of commercial kits for Ab detection based on CF and ID. Urgency for rapid tests (point of care tests) it is highly priority.
**Sporotrichosis:** cases reported across the Americas. *Sporothrix brasiliensis* emerged in Brazil, zoonotic transmission in Brazil and neighboring countries						
In house	Yes	Yes	Yes	No	Yes	Microscopy testing is performed with low sensitivity.Ab detection, variable sensitivity. Limited commercialization of Ab detection kits.LFA for Ab detection development in progress.
Commercial	No	Yes	Yes	No	No	Molecular testing is needed for species identification. Considerable progress in MALDI-TOF identification.
**Chromoblastomycosis and mycetoma:** cases reported across the Americas						
In house	Yes	Yes	No	No	Culture identification	Performance of microscopy is highly variable based on professional laboratory experience and specimen quality.
Commercial	No	Yes	No	No	No	No commercial kits are available for Ab, Ag, and PCR.

*, BSLIII is needed for isolates handling; ±, immunodiagnostics assays; Ab, antibody; Ag, antigen; CF, complement fixation; ID, immunodiffusion; EIA, enzyme immune assay; LFA, lateral flow assay.

Advancements in epidemiology, pathogenesis, diagnosis, and treatment were shared. The discussion delved into the influence of climate change on the geographic distribution of endemic fungi. Evidence was presented illustrating how both global and local climatic variables, particularly anthropogenic changes, can impact the incidence of these mycoses. Emphasis was placed on regions with high endemicity of systemic mycoses, highlighting outbreaks such as coccidioidomycosis in California, blastomycosis in Michigan, histoplasmosis in the Dominican Republic, and the escalating spread of sporotrichosis in Brazil.

The true prevalence of endemic diseases, mortality rates, and their epidemiological characteristics remain uncertain. Strategies to improve routine surveillance of endemic and implantation mycoses in the Americas are needed to address these lingering questions. Drawing from the Brazilian experience in reporting sporotrichosis and other mycoses, Argentina has implemented a mandatory surveillance system for all implantation mycoses and endemic systemic mycoses.

In terms of diagnosis, the challenges associated with culture and identification of certain etiological agents were addressed. The discussion underscored the high sensitivity and specificity of detecting *Sporothrix* in clinical samples through reverse transcriptase PCR and specific PCR, with further species identification facilitated by Matrix-Assisted Laser Desorption/Ionization Time-of-Flight (MALDI-TOF). Additionally, significant strides in sporotrichosis diagnosis were highlighted, including the validation of a lateral flow assay for the detection of anti-*Sporothrix* antibodies.^21^ The use of molecular assays in mycoses, particularly those posing diagnostic difficulties and nonspecific clinical presentations, has showcased advancements, notably demonstrating better diagnostic performance than the use of formalin-fixed paraffin-embedded (FFPE) tissues. Moreover, the exploration of diagnostic algorithms was prompted by the limited utilization of serological tests, which is often hindered by antigen standardization issues, unavailability in certain regions, or the absence of developed serological tests for specific mycoses.

Resistance to antifungals in current clinical practice is increasing but remains largely undocumented in many regions of the Americas. Nonetheless, investigating new antifungal alternatives has unveiled avenues for treatment enhancement. Notably, SUBA-itraconazole exhibits reduced pharmacokinetic variability, fewer adverse events, and effectiveness at lower doses against endemic fungi. Its superior safety profile compared to conventional itraconazole underscores its potential as a therapeutic option.

The effectiveness of treating cats with sporotrichosis using a combination of itraconazole and potassium iodide was shared from a study in Brazil. Emphasis was placed on the importance of implementing thorough cleaning and disinfection measures in the environments where cats reside and are cared for, as well as in the equipment utilized by veterinarians and for transporting infected cats. Additionally, the beneficial use of terbinafine for treating sporotrichosis in pregnant women and individuals with diabetes was underscored.

Discussions also centered on trials involving DNA and RNA vaccines aimed at preventing coccidioidomycosis, with the ambitious goal of safeguarding 50% of the US population by 2030.

On the last day, a diagnostic workshop (WS) on endemic and implantation mycoses was held. This WS covered several aspects of fungal diagnosis. The first part consisted of lectures focusing on the identification of suspected cases, with discussion around clinical cases. These lectures also addressed basic aspects of laboratory biosafety, specimen collection, storage, transport, laboratory analysis, and result interpretation. A patient with a clinical case of mycetoma was presented; this activity allows professionals to see the characteristic disease. The second part was on-hand laboratory training. This training covered conventional testing based on microscopy and key aspects of fungal isolates identification. Furthermore, this WS included an update on rapid non–culture-based testing for the detection of fungal biomarkers (antigens and antibodies).

In conclusion, the lack of surveillance and reportable status of systemic endemic and implantation mycoses in countries in the Americas is largely responsible for the lack of knowledge of the real epidemiology of these mycoses. There is still a need to develop new diagnostic assays, notably immunological and molecular tests. There is an urgent need for rapid and point-of-care diagnostic tools for the detection of biomarkers and genetic materials. Access to and availability of commercial diagnostic kits are critical and can be facilitated by the national registration of products. The need for decentralized testing is another priority, transferring assays from reference centers to hospitals and clinics.

The meeting served as a platform for fostering collaboration among researchers, One Health professionals, and organizations. Collaborative initiatives and partnerships were discussed to strengthen the collective response to endemic and implantation mycoses, promoting information exchange and joint research endeavors.^22^ In summary, the second IMEMA and first ISIM, held in Santiago del Estero, Argentina, proved to be dynamic and collaborative events. By bringing together experts and stakeholders, the conference contributed significantly to the ongoing efforts to combat endemic and implantation mycoses, offering a platform for the exchange of knowledge that will influence research, clinical practice, and public health strategies in the years to come.
